# The Effect of Modified Eggs and an Egg-Yolk Based Beverage on Serum Lutein and Zeaxanthin Concentrations and Macular Pigment Optical Density: Results from a Randomized Trial

**DOI:** 10.1371/journal.pone.0092659

**Published:** 2014-03-27

**Authors:** Elton R. Kelly, Jogchum Plat, Guido R. M. M. Haenen, Aize Kijlstra, Tos T. J. M. Berendschot

**Affiliations:** 1 University Eye Clinic Maastricht, Maastricht University, Maastricht, the Netherlands; 2 Department of Human Biology, Maastricht University, Maastricht, the Netherlands; 3 Department of Pharmacology and Toxicology, Maastricht University, Maastricht, the Netherlands; Cardiff University, United Kingdom

## Abstract

Increasing evidence suggests a beneficial effect of lutein and zeaxanthin on the progression of age-related macular degeneration. The aim of this study was to investigate the effect of lutein or zeaxanthin enriched eggs or a lutein enriched egg-yolk based buttermilk beverage on serum lutein and zeaxanthin concentrations and macular pigment levels. Naturally enriched eggs were made by increasing the levels of the xanthophylls lutein and zeaxanthin in the feed given to laying hens. One hundred healthy volunteers were recruited and randomized into 5 groups for 90 days. Group one added one normal egg to their daily diet and group two received a lutein enriched egg-yolk based beverage. Group three added one lutein enriched egg and group four one zeaxanthin enriched egg to their diet. Group five was the control group and individuals in this group did not modify their daily diet. Serum lutein and zeaxanthin concentrations and macular pigment densities were obtained at baseline, day 45 and day 90. Macular pigment density was measured by heterochromatic flicker photometry. Serum lutein concentration in the lutein enriched egg and egg yolk-based beverage groups increased significantly (p<0.001, 76% and 77%). A strong increase in the serum zeaxanthin concentration was observed in individuals receiving zeaxanthin enriched eggs (P< 0.001, 430%). No changes were observed in macular pigment density in the various groups tested. The results indicate that daily consumption of lutein or zeaxanthin enriched egg yolks as well as an egg yolk-based beverage show increases in serum lutein and zeaxanthin levels that are comparable with a daily use of 5 mg supplements.

**Trial Registration:**

ClinicalTrials.gov NCT00527553

## Introduction

As the world’s population ages, more and more people are affected by age-related macular degeneration (AMD), leading to an increased awareness and interest in the prevention and treatment of this blinding disease [1], [2].

The xanthophylls lutein and zeaxanthin are not endogenously synthesized by the human body and tissue levels therefore depend on dietary intake. These natural compounds found in the bodies of animals, and in dietary animal products, are ultimately derived from plant sources in the diet, mainly from dark green leafy plants. The xanthophylls lutein, zeaxanthin and meso-zeaxanthin are naturally occurring macular pigments, giving the fovea its’ yellowish color [3]. These specific xanthophylls do not function in the mechanism of sight, since they cannot be converted to retinal. These xanthophylls have anti-oxidative and blue-light filtering properties [4]–[6]. Recent evidence suggests that they also have anti-inflammatory properties thereby reducing immune-mediated damage to the macula [7], [8].

It has been shown that increased consumption of lutein and zeaxanthin markedly increases the concentration in blood, which can subsequently lead to an increase in macular pigment levels [9]–[12]. Accumulation of these xanthophylls in the retina is considered to play a role in the prevention of age-related macular degeneration. In line with these assumptions, epidemiological studies have indeed shown that subjects with the highest dietary intake of lutein and zeaxanthin have a lower prevalence and incidence of age-related macular degeneration [13], [14]. Lower macular xanthophyll levels have been associated with an increased risk of AMD progression [15], [16]. Altogether, these findings have prompted many individuals to take supplements containing both lutein and zeaxanthin as a preventive strategy against AMD.

Egg yolks are an important natural dietary source of lutein and zeaxanthin and their concentration can easily be enhanced via the feed given to laying hens [17]–[19].Within eggs, lutein and zeaxanthin are packed into lipid matrixes. Studies in humans have shown that these lipid-rich matrixes result in a relatively higher xanthophyll bioavailability as compared to other dietary sources such as spinach [20].

In this study we investigated whether daily intake of lutein or zeaxanthin enriched eggs could lead to increased serum values, to provide an alternative to the daily consumption of supplements. We also included an egg-yolk based beverage, that was developed from a practical point of view in terms of providing possibilities to maintain compliance. Furthermore, an advantage of a beverage over eggs is the fact that it is much better suited for future double blind, placebo controlled studies since color and taste of such beverages can be adjusted. Our study shows that intake of lutein enriched eggs can raise circulating lutein and zeaxanthin levels to values that are comparable with taking 5 mg (pill) supplements.

## Subjects and Methods

The protocol for this trial and supporting CONSORT checklist are available as supporting information; see Checklist S1 and Protocol S1.

### Ethics statement

This study was conducted according to the declarations of Helsinki and was approved by the Medical Ethical Committee of the University Hospital of Maastricht. Written informed consent was obtained from all participants.

### Subjects

For this study one hundred subjects were recruited through local newspapers and posters at the university and hospital buildings. Healthy individuals, 18 years and older were eligible for participation. Exclusion criteria were diabetes, having heart disease, lipid metabolic diseases, AMD in both eyes (at least the eye studied in the trial had to be healthy), ocular media opacity or other ocular diseases. Most studies that have investigated the relationship between macular pigment optical density and cigarette smoking have reported an inverse association between these two. In order to avoid possible confounding we therefore chose not to include smokers. Furthermore, individuals taking supplements containing lutein and/or zeaxanthin in the past six months, and those with a body mass index > 30 kg/m^2^ were excluded. Finally, only subjects with a macular pigment optical density (MPOD) score below 0.55 were included. Recruitment started September 2007. Data collection ended February 2008.

### Diet and design

Lutein and zeaxanthin enriched eggs as well as the lutein egg yolk beverage were obtained from Newtricious (Oirlo, The Netherlands). These eggs were produced by feeding laying hens with feed enriched with natural sources of lutein and or zeaxanthin. The exact composition of the feed cannot be provided due to proprietary reasons of the manufacturer but did not exceed the EU regulation concerning the maximal xanthophyll level of 80 ppm. The lutein egg yolk beverage was based on traditionally prepared buttermilk drink, indicating that it consisted of the liquid remaining after the manufacturing of cheese and butter (churning) [21]. Vanilla sugar was added to increase palatability (5.0 g per 100 mL). All appropriate food safety standards were applied to the ingredients, the processing and the packaging of the investigational products.

Subjects were randomly allocated into one of five groups. Group one received 1 normal egg a day, group two received a beverage prepared from a lutein enriched egg yolk (one yolk per day), group three received one lutein enriched egg per day, and group four consumed a zeaxanthin enriched egg a day. Finally, group five was the control group, whereby individuals did not modify their daily diet. The egg groups were double blinded; this was for obvious reasons not possible for the egg beverage group. Eggs were analyzed on carotenoid content throughout the study to ensure consistency. Table 1 shows lutein and zeaxanthin concentration of the different egg/egg products used in this study. Subjects were tested on day one when they started with the trial, at day 45 and at the end of the study on day 90. They were asked not to make any major modifications to their diet except for the addition of the egg/egg yolk containing beverage to their daily lunch or dinner for the duration of the study.

**Table 1:** Lutein and zeaxanthin content of egg yolks (mean ± standard deviation).

	Egg yolk concentration	
Egg type	Lutein	Zeaxanthin
Non enriched	167.8±8.7 μg/yolk	85.0±1.7 μg/yolk
Lutein enriched	921.4±105 μg/yolk	137.3±14.0 μg/yolk
Zeaxanthin enriched	174.3±14.5 μg/yolk	487.3±31.0 μg/yolk
Lutein beverage^1^	970	340

1 Based on one egg yolk. Data are from a homogenized sample. Measurement error was 5.7 μg for lutein and 2 μg for zeaxanthin.

### Serum analysis

Fasting blood samples were taken using 10 mL serum tubes (Becton, Dickinson and Company, Franklin Lakes, NY, USA). These were left for at least 30 minutes before they were centrifuged for 30 minutes at 4°C and 2.000 g, divided into 500 μL portions, snap frozen and stored at –80°C for later analysis as a single batch. Lutein and zeaxanthin concentrations were analyzed using high performance liquid chromatography (HPLC) [22]. Briefly, on the day of analysis, the samples were thawed and mixed well. Samples were deproteinized by adding a 500 μL sample to 500 μL ethanol. The samples were mixed and allowed to stand for 15 minutes at room temperature to complete the precipitation of proteins. The carotenoids were subsequently extracted by adding 1.0 mL n-hexane. After centrifugation for 10 minutes at 4°C and 3.000 g, 0.5 mL of the upper hexane layer was evaporated to dryness under a stream of nitrogen. The residue was dissolved in 0.5 mL of a mixture of methanol, acetonitril (1∶1) and dichloromethane and subsequently analyzed by HPLC. Separation was obtained on a C18 reversed-phase column, thermostatically controlled at 30°C. The samples were eluted by use of a mobile phase consisting of methanol, acetonitril, 2-propanol and water at a flow rate of 1.5 mL/min. Detection was performed with a diode array UV detector at 450 nm. Quantification was carried out by including commercially available lutein and zeaxanthin as a standard (Sigma-Aldrich, St Louis, USA).

### Macular pigment

The amount of macular pigment is determined by measuring its absorbance, the macular pigment optical density (MPOD). This dimensionless parameter is defined as the logarithmic ratio of the light falling upon a material, to the light transmitted through a material.

MPOD was determined using the principle of heterochromatic flicker photometry (QuantifEYE; Topcon, Newbury, UK) [23]. In short, observers view a target composed of two light emitting diodes (blue, 470 nm and green, 540 nm) flickering in counter-phase. Initially the luminance of the green light is higher than that of the blue. The initial temporal frequency is above the normal critical flicker fusion frequency (60 Hz) and is reduced at 6 Hz/sec. The subject fixates on the target and presses a button when flicker is detected. The luminance ratio of blue and green is then changed, incrementing blue and decrementing green. The temporal frequency is re-set to 60 Hz and again ramped down at 6 Hz/sec, until the subject detects flicker and presses the response button. This cycle continues for a series of blue-green luminance ratios until a V shaped function is obtained with a clear minimum that corresponds to the equalization of the blue and green luminance. This process of detecting flicker for a series of blue-green luminance ratios is then repeated for peripheral viewing at 6 degrees eccentricity, and again a V shaped curve is obtained which provides a minimum for the periphery. The MPOD is then calculated according to the formula MPOD  =  Log[Lc/Lp], where Lc and Lp are the luminances of the blue light at the minimum for central and peripheral viewing, respectively.

### Power calculation and randomization

Intake of 10 mg lutein from supplements induces a 5% monthly increase in MPOD [9]. Although the amount of lutein per egg is much less, the bioavailability is higher [20]. We therefore anticipated a 3% monthly increase in MPOD, resulting in a 9% MPOD increase over the study period. The repeatability of the QuantifEYE is 11.7% [23]. With α = 0.05 and β = 0.1 this yielded 18 subjects per group [24]. Taking into account a 10% dropout we included 20 subjects per group. The random allocation sequence was generated by AK using Research Randomizer [25].

### Statistical analyses

Statistical analysis was performed using SPSS 21.0.0.1. Differences in gender distribution over the experimental groups were tested using the Pearson Chi-square test, while differences in age and serum concentrations between groups was evaluated using ANOVA. To analyze serum lutein/zeaxanthin and MPOD, i.e. changes in time and possible differences between groups, we performed a Linear Mixed Models analysis (LMM) with subject as grouping factor, and gender, diet, time and the interaction term of the latter two as covariates. The LMM procedure expands the general linear model so that the data are permitted to exhibit correlated and non-constant variability. The LMM analysis, therefore, provides the flexibility of modeling not only the means of the data but their variances and covariances as well. LMM handle data where observations are not independent. That is, LMM correctly models correlated errors, whereas procedures in the general linear model family usually do not. Since we used subject as grouping factor, we take into account individual differences in baseline values. The model does assume similar linear changes in time for all subjects. P-values were considered significant if P<0.05. For post-hoc test we applied the Bonferroni correction. Results are shown as mean ± standard deviation.

## Results

**Figure 1:**
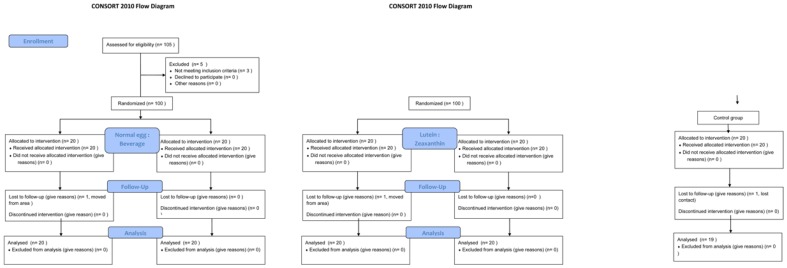
Flow diagram of the participants in the study.

In total, 97 subjects completed the entire study. Two dropped out because they moved out of the area and one for unknown reasons (see Figure 1). Table 2 presents baseline characteristics of the participants. There were no statistically significant differences between the five groups for age (P = 0.34), gender (P = 0.99), serum zeaxanthin (P = 0.48) and MPOD (P = 0.60). There was a significant difference between the groups for serum lutein at baseline (P = 0.030). A post-hoc analysis showed that the lutein egg group had significantly higher serum baseline levels. All other groups were comparable.

**Table 2:** Baseline characteristics of study subjects (mean ± standard deviation).

	Age	Gender (m/f)	Lutein (ng/ml)	Zeaxanthin (ng/ml)	MPOD^1^
Normal Egg	53±12	9/11	216±106	27.8±19.3	0.31±0.14
Lutein beverage	43±16	8/12	174±69	23.5±14.5	0.38±0.12
Lutein Egg	45±19	8/12	290±213	27.5±16.4	0.32±0.12
Zeaxanthin Egg	48±17	9/11	217±104	29.2±23.2	0.35±0.14
Control	44±16	9/11	180±51	20.4±10.2	0.34±0.15
P-value	0.34	0.99	0.030	0.48	0.60

1 MPOD Macular Pigment Optical Density.

P-values are from an ANOVA analysis comparing the different groups.

Supplementation with lutein enriched eggs caused a 76% increase in serum lutein concentrations while the egg-yolk based beverage caused a comparable increase of 77% (P<0.001, paired T-test, Table 3). Daily consumption of a normal egg or a zeaxanthin enriched egg also led to an increase of the serum lutein levels of 16 and 18% respectively, but these changes did not reach significance (P>0.29). This was confirmed by an LMM analysis using the serum lutein levels as dependent, and with diets, gender and time and their interaction terms as covariate. A significant effect was observed for time (P<0.001), gender (P = 0.012, women having on average 61 ng/ml higher lutein level than men) as well as an interaction between diet and time on serum lutein level (P<0.001). This interaction was seen for both the lutein enriched eggs as for the lutein enriched egg-yolk based beverage groups as compared to the control group. Table 4 shows the β-coefficients of the LMM analysis and their P-values, with the control group as the reference. No difference was observed concerning the increase in serum lutein level between the lutein enriched egg and the lutein enriched egg-yolk based beverage group.

**Table 3:** Serum lutein concentrations (ng/ml, mean ± standard deviation) during study follow up in males and females separately and combined.

Lutein	Baseline				Midpoint				Endpoint			
	Both	Male	Female	P^2^	Both	Male	Female	P^2^	Both	Male	Female	P^2^
Normal Egg	216±106	193±76	235±126	0.40	239±111	205±79	266±129	0.23	235±93	212±54	255±117	0.33
Lutein beverage	174±69	137±40	198±75	0.048	284±126	210±85	334±126	0.026	310±135	219±75	371±133	0.009
Lutein Egg	290±213	213±96	334±251	0.24	438±196	336±114	497±212	0.082	465±201	389±125	509±227	0.22
Zeaxanthin Egg	217±104	215±112	219±102	0.93	232±125	234±155	230±99	0.95	248±118	235±130	260±112	0.67
Control	180±51	174±48	185±56	0.66	188±72	190±75	186±72	0.91	177±55	169±59	184±53	0.55
P-value^1^	0.030	0.27	0.11		<0.001	0.082	<0.001		<0.001	0.001	<0.001	

1 P-values are from an ANOVA analysis comparing the different groups at each time point.

2 P-values for the difference between male and female subjects.

**Table 4:** β-coefficients of the Linear Mixed Model (LMM) analyses, their P-values and confidence intervals.

	Lutein			Zeaxanthin			MPOD		
	β	P-Value	CI	β	P-Value	CI	β	P-Value	CI
Intercept	149.5	<0.001	89.1 – 210.0	25.0	<0.001	11.3 – 38.6	0.32	<0.001	0.25 – 0.38
Diet:Normal Egg	36.5	0.35	–40.5 – 78.6	7.0	0.44	–10.8 – 24.7	–0.021	0.62	–0.10 – 0.06
Diet:Lutein beverage	1.5	0.97	–75.6 – 78.6	2.8	0.76	15.0 – 20.6	0.003	0.93	–0.08 – 0.08
Diet:Lutein Egg	121.6	0.003	43.4 – 199.7	7.7	0.4	–10.4 – 25.7	–0.017	0.68	–0.99 – 0.06
Diet:Zeaxanthin Egg	35.4	0.37	–42.7 – 113.4	23.4	0.011	5.4 – 41.4	–0.006	0.89	–0.09 – 0.08
Diet:Control	0			0			0		
Gender:Female	61.3	0.012	13.6 – 109.0	–5.1	0.3	–14.8 – 4.6	0.022	0.36	–0.03 – 0.07
Gender:Male	0			0			0		
Time	–0.23	0.87	–2.9 – 2.5	0.53	0.36	–0.6 – 1.7	0.002	0.47	–0.003 – 0.007
Time*Diet:Normal Egg	2.2	0.26	–1.6 – 6.1	1	0.23	–0.6 – 2.6	0.002	0.58	–0.006 –0.010
Time*Diet:Lutein beverage	11.6	<0.001	7.8 – 15.4	1.29	0.12	–0.3 – 2.0	–0.004	0.34	–0.011 – 0.004
Time*Diet:Lutein Egg	14.9	<0.001	11.0 – 18.7	1.58	0.059	–0.05 – 3.2	0.004	0.3	–0.004 – 0.012
Time*Diet:Zeaxanthin Egg	2.8	0.16	–1.1 –6.7	7.65	<0.001	6.0 –9.3	0	0.94	–0.008 – 0.007
Time*Diet:Control	0			0			0		

The serum zeaxanthin level increased markedly (430%) following consumption of zeaxanthin enriched eggs, whereas no significant changes were observed in the other four groups (Table 5). A LMM analysis with serum zeaxanthin levels as dependent, and with diets and gender as factor and time, and the interaction between diet and time as covariates revealed a significant effect for time (P<0.001) and the interaction term diets and time (P<0.001). This interaction was seen for the zeaxanthin enriched egg group (P<0.001) as compared to the control groups (see Table 4). A gender effect could not be detected.

**Table 5:** Serum zeaxanthin concentrations (ng/ml, mean ± standard deviation) during study follow up.

Zeaxanthin	Baseline				Midpoint				Endpoint			
	Both	Male	Female	P^2^	Both	Male	Female	P^2^	Both	Male	Female	P^2^
Normal Egg	27.8±19.3	37.2±23.4	20.1±11.1	0.45	40.9±23.4	51.5±27.1	32.2±16.4	0.63	45.6±25.3	53.7±30.6	38.4±18.1	0.20
Lutein beverage	23.5±14.5	20.3±7.4	25.7±17.8	0.42	37.8±19.8	28.1±8.9	44.3±22.6	0.07	45.4±18.1	37.6±11.4	50.6±20.3	0.12
Lutein Egg	27.5±16.4	31.3±18.2	25.3±15.7	0.45	45.9±16.2	52.4±16.7	42.1±15.3	0.19	52.8±23.3	62.4±27.5	47.3±19.6	0.18
Zeaxanthin Egg	29.2±23.2	31.0±29.3	27.7±17.6	0.77	127.8±65.3	129.9±77.1	125.8±56.2	0.89	127.5±54.3	125.8±77.1	128.9±24.8	0.91
Control	20.4±10.2	23.7±10.3	17.75±9.9	0.21	28.8±22.4	36.1±31.1	22.8±9.8	0.20	26.8±10.6	29.9±13.5	24.18±7.2	0.24
P-value^1^	0.48	0.43	0.49		<0.001	<0.001	<0.001		<0.001	<0.001	<0.001	

1 P-values are from an ANOVA analysis comparing the different groups at each time point.

2 P-values for the difference between male and female subjects.

No MPOD changes could be detected over time during the 90 day’s trial period (Table 4 and 6).

**Table 6:** Macular pigment optical density (MPOD) during study follow up.

MPOD	Baseline				Midpoint				Endpoint			
	Both	Male	Female	P^2^	Both	Male	Female	P^2^	Both	Male	Female	P^2^
Normal Egg	0.31±0.14	0.27±0.09	0.34±0.17	0.25	0.35±0.18	0.30±0.11	0.40±0.22	0.25	0.35±0.22	0.25±0.13	0.45±0.25	0.04
Lutein beverage	0.38±0.12	0.37±0.14	0.38±0.11	0.96	0.32±0.15	0.34±0.15	0.30±0.15	0.54	0.32±0.16	0.33±0.20	0.32±0.14	0.96
Lutein Egg	0.32±0.12	0.27±0.11	0.35±0.12	0.18	0.42±0.23	0.33±0.06	0.48±0.27	0.18	0.36±0.16	0.32±0.16	0.38±0.17	0.44
Zeaxanthin Egg	0.35±0.14	0.29±0.12	0.40±0.13	0.07	0.31±0.16	0.25±0.15	0.37±0.15	0.81	0.36±0.21	0.42±0.27	0.31±0.14	0.33
Control	0.34±0.15	0.41±0.16	0.28±0.14	0.08	0.36±0.22	0.38±0.18	0.35±0.26	0.78	0.35±0.17	0.41±0.17	0.29±0.15	0.11
P-value^1^	0.60	0.37	0.08		0.38	0.37	0.35		0.96	0.24	0.31	

1 P-values are from an ANOVA analysis comparing the different groups at each time point.

2 P-values for the difference between male and female subjects.

## Discussion

Daily consumption of lutein enriched eggs caused a 76% increase in serum lutein concentration. A similar increase of 77% was observed in the group taking a lutein enriched egg beverage. Consumption of zeaxanthin enriched eggs caused a marked 430% increase in serum zeaxanthin concentration. Our data are in agreement with earlier studies that showed that the daily consumption of eggs results in significantly increased serum xanthophyll levels [18], [20], [26]–[28].However, consumption of lutein or zeaxanthin enriched egg yolks led to a higher increase in xanthophylls blood levels compared to studies using “normal” eggs. Goodrow *et al*. for instance found a 26% and 38% increase in serum lutein and zeaxanthin concentrations following consumption of normal non-enriched eggs, that contain less lutein and zeaxanthin (143±28 and 94±18 μg/yolk) as compared to the enriched eggs used in our study [18]. In our non-enriched egg group (Table 3 and 5) we observed an increase of 16% in serum lutein and a 100% increase in serum zeaxanthin concentrations. Handelman *et al*. found a 28% and 142% increase using 1.3 egg yolk containing 292±117 μg/yolk of lutein and 213±85 μg/yolk of zeaxanthin for 4.5 weeks [26].

Most interest concerning xanthophylls in eggs in the past was related to consumer’s wishes concerning egg yolk color. This can be achieved by raising the amount of natural or synthetic xanthophylls in chicken feed. It should be noted that the enriched eggs used in our study were obtained by providing the laying hens with a feed containing a higher amount of natural sources of xanthophylls derived from maize and marigold sources.

There is a growing awareness that individuals can modulate their risk of developing AMD by enhancing their intake of xanthophylls like lutein and zeaxanthin. They can either chose to do so by taking lutein and/or zeaxanthin containing supplements (pills) or by changing their diet, including functional foods. Lutein or zeaxanthin enriched eggs can be seen as a functional food to improve the xanthophyll status of an individual. In Europe, many elderly would chose to not take pills but to improve their lifestyle. Our investigations are aimed at proving the feasibility of such an approach, whereby we argued that consumption of xanthophyll enriched eggs could be a natural alternative for a pill. Xanthophyll content of eggs not only depends on the feed given to laying hens, but is also dependent on the husbandry system [29], [30]. Organic eggs were shown to have the highest lutein content (mean: 1,764 ug/100 g egg yolk), whereas caged eggs have the lowest level (mean: 410 ug/100 g egg yolk ) [30]. Organic chickens obtain their xanthophylls from the grass and herbs in the outdoor run and due to the fact that outdoor run coverage and the outdoor run use by the hens is extremely variable, the xanthophyll content in eggs from this husbandry system has a wide range. The amount of lutein in the eggs used in our study can be maintained fairly constant due to the use of a well-defined feed. The obtained lutein content in the eggs used in our study contained approximately 4,600 ug of lutein per 100 g of egg yolk, which is almost threefold higher than the levels present in organic eggs. The zeaxanthin levels reached in our eggs (2,435 ug/ 100 g egg yolk) was also much higher than observed in organic eggs (1,021 ug/100 g egg yolk).

An important aspect of using eggs or egg yolks as a lutein source relates to the suggested elevated bioavailability [20]. It is well known that lutein depends on a lipophilic environment for optimal gastrointestinal uptake [31]. The change in serum lutein concentrations we observed following daily consumption of an egg yolk containing one mg of lutein per day, was comparable to the increase observed in a study using a five mg lutein supplement [32]. The finding that the zeaxanthin supplemented group had a 430% increase in serum zeaxanthin concentration compared to the lutein supplemented group which had a 76% increase in serum lutein concentration may be due to the fact that the zeaxanthin enriched egg had a relatively high zeaxanthin level as compared to the other dietary sources taken by our participants. This is evident in view of the fact that baseline blood levels of zeaxanthin were much lower as compared to the lutein blood level. We did not asses the diets taken by our participants and further studies are needed to address this issue.

Safety issues of this study included the fact that an increased egg consumption can alter circulating lipoprotein levels and that the use of xanthophyll enriched eggs could result in a higher circulating level of lutein and zeaxanthin in our volunteers. Eggs are a concentrated source of cholesterol [33] and dietary cholesterol increases low-density lipoprotein cholesterol (LDL-C) concentrations [34]. To address these possible adverse side effects we compared serum lipoprotein levels between the different groups. None of the individuals participating in the study attained a lipoprotein level that was considered as abnormal. The data that are presented in a separate paper showed that the rise in serum LDL-C concentrations was less pronounced when egg yolk was incorporated into the buttermilk drink, suggesting that fractions in the buttermilk may influence dietary cholesterol absorption [35].

We did not perform any additional tests to address possible safety issues related to an increased lutein or zeaxanthin level associated with the consumption of the enriched eggs used in our project since this has been covered in detail in earlier studies [36], [37]. Short-term and long-term toxicity profile of lutein was studied in young adult Wistar rats [38]. Administration of up to 400 mg/kg body weight of lutein did not produce any mortality, change in body weight, food consumption pattern, organ weight, and other adverse side reactions. It also did not alter hepatic or renal function, and did not produce any change in the hematological parameters or lipid profile. Histopathological analysis of the organs supported the absence of toxicity of lutein. A similar study was undertaken by Ravikrishnan *et al*. [36]. In comparison with a control group, they also found no treatment-related changes in clinical observations, ophthalmic examinations, body weights, body weight gains, feed consumption, and organ weights. No toxicologically relevant findings were noted in urinalysis, hematology or clinical biochemistry parameters. Terminal necropsy did not reveal any treatment-related gross or histopathology findings. The no observed-adverse-effect level for their lutein/zeaxanthin concentrate was determined as 400 mg/kg bw/day, the highest dose tested. These levels are far higher than the dose used in our study which is equivalent to a 5 mg lutein containing daily supplement per individual.

Others studied acute and subacute toxicity of lutein in lutein-deficient mice [39]. Compared to a control (peanut oil without lutein) group, they found no treatment-related toxicologically significant effects of lutein in clinical observation, ophthalmic examinations, body, and organ weights. Further, no toxicologically significant findings were eminent in hematological, histopathological, and other clinical chemistry parameters. Further, in an oral sub acute toxicity study, the no-observed-adverse-effect level for lutein was determined as 1000 mg/kg/day, the highest dose tested, which as mentioned above is several orders of magnitude higher than our doses.

Various independent clinical studies assessing retinal electrophysiological functions following the intake of lutein or zeaxanthin containing supplements did not show adverse effects and even reported an improved retinal function [40]–[43].

We could not detect a significant change in the macular pigment optical density during our trial among the various groups tested. We believe that this is an absence of evidence instead of evidence of absence of an effect. It is probably due to the fact that our study was carried out during a relatively short time period. Xanthophyll supplementation leads to a rapid response in circulating blood levels and a subsequent slow increase in macular pigment levels. Bone and Landrum showed that with supplementation MPOD tends to exhibit a linear increase in time [32], in particular in subjects proficient in heterochromatic flicker photometry. However, they also showed a great variation in the standard error of the slope of this linear increase, which implies that the fact that we did not achieve a measurable increase in the MPOD values is due to a small sample size and that a longer intervention period is needed to reach significance.

Participants were asked not to change their habitual diet, level of physical exercise, or use of alcohol throughout the study, although we did not assess their diet. On the other hand, participants were aware of the nature and background of the study and it cannot be excluded that this may have inadvertently affected their behavior. However, due to the strictly randomized placebo controlled nature of the trial this should have affected all groups. Medical ethical committees insist that subjects are adequately informed about the nature of clinical trials and one should be aware of the fact that this may introduce confounding circumstances. In the study we did not address possible confounders and to rule this out a larger sample size will be needed.

Using supplements varying from five to twenty mg of lutein, others have shown that serum lutein concentration increased rapidly during the first two–three weeks of supplementation, whereafter it reached a plateau up to the end of the supplementation period [32]. Although we only included three time points in our study, it appears that consumption of lutein enriched eggs causes a continuous increase in serum lutein levels during the whole trial period of 90 days, although less fast in the second half of the study. On the other hand, the changes in serum zeaxanthin levels following the daily consumption of a zeaxanthin enriched egg were more in line with results as shown earlier by others [32]. Serum zeaxanthin levels in our study leveled after week six. Since our first measurement was at week six, it is possible that maximum levels were already reached at an earlier time point.

It should be kept in mind that the present study does not provide evidence to support the fact that modified eggs or egg-yolk based beverages constitute a preventive or curative treatment of any form of age related macular degeneration or maculopathy.

In summary, we found significant increases in serum lutein and zeaxanthin concentration following consumption of enriched eggs providing one mg lutein/day, which was comparable with a daily use of five mg supplements. An egg yolk-based beverage showed similar results. Our study provides support for the concept that an adequate xanthophyll status can be achieved by the intake of naturally enriched functional foods.

## Supporting Information

Checklist S1 CONSORT checklist.
(PDF)

Protocol S1 Trial protocol.
(PDF)
