# Supplementary material for: The Effect of Modified Eggs and an Egg-Yolk Based Beverage on Serum Lutein and Zeaxanthin Concentrations and Macular Pigment Optical Density: Results from a Randomized Trial
Source: PLoS One. 2014 Mar 27;9(3):e92659. doi: 10.1371/journal.pone.0092659 (PMC3968018; doi:10.1371/journal.pone.0092659)
Supplement: Protocol S1 — Trial protocol. (PDF) [file pone.0092659.s002.pdf]

RESEARCH PROTOCOL

The effect of modified eggs and egg products on the measurable macular pigment in healthy test subjects.

TABLE OF CONTENTS

1. INTRODUCTION AND RATIONALE 8
2. OBJECTIVES 10
3. STUDY DESIGN 11
4. STUDY POPULATION 12
4.1 Population (base) 12
4.2 Inclusion criteria 12
4.3 Exclusion criteria 12
4.4 Sample size calculation 12
4.5 Investigational product/treatment 13
4.6 Use of co-intervention (if applicable) 13
4.7 Escape medication (if applicable) 13
5. INVESTIGATIONAL MEDICINAL PRODUCT 14
5.1 Name and description of investigational medicinal product 14
5.2 Summary of findings from non-clinical studies 14
5.3 Summary of findings from clinical studies 14
5.4 Summary of known and potential risks and benefits 14
5.5 Description and justification of route of administration and dosage 14
5.6 Dosages, dosage modifications and method of administration 15
5.7 Preparation and labeling of Investigational Medicinal Product 15
5.8 Drug accountability 15
6. METHODS 17
6.1 Study parameters/endpoints 17
6.1.1 Main study parameter/endpoint 17
6.1.2 Secondary study parameters/endpoints (if applicable) 17
6.1.3 Other study parameters (if applicable) 17
6.2 Randomisation, blinding and treatment allocation 17
6.3 Study procedures 17
6.4 Withdrawal of individual subjects 19
6.4.1 Specific criteria for withdrawal (if applicable): 19
6.5 Replacement of individual subjects after withdrawal 19
6.6 Follow-up of subjects withdrawn from treatment 19
6.7 Premature termination of the study 19
7. SAFETY REPORTING 21
7.1 Section 10 WMO event 21
7.2 Adverse and serious adverse events 21
7.3 Follow-up of adverse events 22
7.4 Data Safety Monitoring Board (DSMB) 22
8. STATISTICAL ANALYSIS 23
9. ETHICAL CONSIDERATIONS 24
9.1 Regulation statement 24
9.2 Recruitment and consent 24

9.3 Objection by minors or incapacitated subjects (if applicable) 24
9.4 Benefits and risks assessment, group relatedness 24
9.5 Compensation for injury 25
9.6 Incentives 25
10. ADMINISTRATIVE ASPECTS AND PUBLICATION 26
10.1 Handling and storage of data and documents 26
10.2 Amendments 26
10.3 Annual progress report 26
10.4 End of study report 26
10.5 Public disclosure and publication policy 27
11. REFERENCES 28

LIST OF ABBREVIATIONS AND RELEVANT DEFINITIONS

**ABR**	**ABR form (General Assessment and Registration form) is the application form that is required for submission to the accredited Ethics Committee (ABR = Algemene Beoordeling en Registratie)**
**AE**	**Adverse Event**
**AR**	**Adverse Reaction**
**CA**	**Competent Authority**
**CCMO**	**Central Committee on Research Involving Human Subjects**
**CV**	**Curriculum Vitae**
**DSMB**	**Data Safety Monitoring Board**
**EU**	**European Union**
**EudraCT**	**European drug regulatory affairs Clinical Trials GCP Good Clinical Practice**
**IB**	**Investigator's Brochure**
**IC**	**Informed Consent**
**IMP**	**Investigational Medicinal Product**
**IMPD**	**Investigational Medicinal Product Dossier**
**METC**	**Medical research ethics committee (MREC); in Dutch: medisch ethische toetsing commissie (METC)**
**(S)AE**	**Serious Adverse Event**
**SPC**	**Summary of Product Characteristics (in Dutch: officiële productinfomatie IB1-tekst)**
**Sponsor**	**The sponsor is the party that commissions the organisation or performance of the research, for example a pharmaceutical company, academic hospital, scientific organisation or investigator. A party that provides funding for a study but does not commission it is not regarded as the sponsor, but referred to as a subsidising party.**
**SUSAR**	**Suspected Unexpected Serious Adverse Reaction**
**Wbp**	**Personal Data Protection Act (in Dutch: Wet Bescherming Persoonsgevens)**
**WMO**	**Medical Research Involving Human Subjects Act (Wet Medisch-wetenschappelijk Onderzoek met Mensen**

SUMMARY

Rationale: Age-related macula degeneration (AMD, encompassing both dry and wet form), the late stage of Age-related maculopathy (ARM), is the leading cause of blindness in many developed countries [1-6] in older persons (usually over 60 years of age). Visual compromise rises exponentially after age of 70 [7] with a 5-year incidence of around 1%. Studies have shown a possible protective effect of lutein on progression of AMD, where visual acuity improves after increased lutein intake [8]. The incidence of bilateral AMD in persons with unilateral late ARM observed over a period of 10 years of over 50% with a 2.1-2.8% overall incidence in study population [9].

Blue light hazard (excitation peak 440nm) was shown to have major impact on photoreceptor and RPE function inducing photochemical damage and cellular apoptosis, leading to retinal degeneration in animal study [10].The current believe is that lutein accumulated in the macular region helps in the prevention of blindness by absorbing blue light and protecting the retina from oxidative stress [11]. With the lipid matrix of the egg yolk being a proven vehicle for the efficient absorption of dietary lutein [12], it might be possible to increase plasma levels of lutein to therapeutic levels and control or prevent AMD. This we hope will be accomplished by means of filtering out harmful blue light and the scavenging of free radicals by lutein and zeaxanthin.

Objective:

1. Assess any change in macular pigmentation due to lutein and zeaxanthin enriched egg consumption. Our hypothesis is that eggs are very effective at increasing macular pigment optical density by increasing the amount of lutein and zeaxanthin in the *macula lutea*.
2. Assessing changes in retinal morphology, visual acuity and contrast sensitivity. We will also analyze lipid profile in its relation to daily egg consumption.

This study uses some of measuring techniques mentioned in protocol id MEC 06-1-052 where tropicamide is also used and also measures macular pigment optical density.

Study design: Randomized placebo controlled interventional trail, where subjects divided into 5 groups, will be consuming either no eggs, normal eggs (lutein: 0.168 ± 0.08, zeaxanthin 0.085 ± 0.0017), eggs enriched with lutein (lutein: 0.921 ± 0.106, zeaxanthin: 0.137 ± 0.014), eggs enriched with zeaxanthin (lutein: 0.174 ± 0.014, zeaxanthin: 0.487 ± 0.031), or egg product made from the lutein enriched eggs. The omega-3 concentration is the same for all eggs and is 200 ± 10mg. Subjects will pick up their test product (if applicable) at every control session and twice between every control session they will have to come to the Human Biology distribution department to receive additional products. The control group

will be asked to not consume any addition eggs to what they normally do, and everyone will be asked not to make any substantial change to their normal diet.

Study population: Healthy human volunteers, 18 years and older.

Intervention (if applicable): Each individual will consume one egg or egg product daily for 90 days. The trial group will receive the modified eggs or egg product while the control group will receive conventional egg.

In egg (mg/egg)	Lutein<br>Mg per egg yolk		Zeaxanthine<br>Mg per egg yolk	
	mean	St dev	mean	St dev
Control	0,16778	0,008655	0,08504	0,001692
Lutein	0,921422	0,105767	0,137318	0,013953
Zeaxanthine	0,1743	0,014454	0,487303	0,031019

Main study parameters/endpoints:

- Macular pigment optical density

Nature and extent of the burden and risks associated with participation, benefit and group relatedness: Subjects have to change their diet to the extent that they will have to include one egg or egg product daily to their lunch. Depending on the group they are enrolled in, these eggs or egg products will be made from conventional eggs or modified eggs. Those consuming conventional eggs or egg product will not at any time be exposed to any additional risk compared to their normal egg consumption. There is to date no evidence that indicate that daily egg consumption will increase the risk of cardio vascular disease, as daily egg consumption may result in an increase in cholesterol levels [13, 14]. Those consuming the modified eggs or egg products will be ingesting a higher amount of lutein, zeaxanthin and omega-3 fatty acid than they would otherwise do. These have however been proven safe for consumption at the levels we will be testing [15-17].

The mean visual acuity test using the Early Treatment Diabetic Retinopathy Study (ETDRS)-chart and the contrast sensitivity test using the Pelli Robson chart are also non-invasive. These too will require time and cooperation of the test subject. Further measurements will be performed using various techniques. These have all been used in previous research or clinical settings and proven safe for use on subjects. The Scanning Laser Ophthalmoscope (SLO)[18, 19], Optical Coherence Tomography (OCT, being used daily at our own ophthalmology department), Heterochromatic Fluorescence Photometry (HFP) [20], and the Reflectometer (RM) [21] have all been proven safe. However, all these measurements will take time to be performed and the patient will have to sit still during the whole measuring procedure last approximately 50 minutes per session. All these test will be performed at all three visits.

The same applies to the blood sampling (approximately 10 minutes). This will also be performed 3 times, once every visit. There is the possibility of the occurrence of a haematoma at the insertion site but this is a self-limiting and non life threatening situation. It's extend can, if need be, be further limited by adding extra compression at the site. If and when there is a haematoma, it will be cleaned by the body's own immune system.

The questionnaire will be taken once, at start of the trial, consisting of 15 items.
A nutrition diary will be kept for three different days during the whole trial.

For some of the measurements the pupil will be dilated using Tropicamide 0.5%, this is also a standard practise at our ophthalmology department with only sporadic, and treatable side effects (acute angle-closure glaucoma in 0.03% of all subject being dilated) [22, 23]. This dilation will be done at every visit.

	**Day 1**	**Day 45**	**Day 90**
**Visual Acuity test**	**X**	**X**	**X**
**Contrast sensitivity**	**X**	**X**	**X**
**SLO**	**X**	**X**	**X**
**HFP**	**X**	**X**	**X**
**OCT**	**X**	**X**	**X**
**Reflectometer**	**X**	**X**	**X**
**Blood Sampling**	**X**	**X**	**X**
**Questionnaire**	**X**	**-**	**-**

1. INTRODUCTION AND RATIONALE

Age-related macula degeneration (AMD, both wet and dry form) is one of the leading causes of blindness in the elderly (60 years or older) in many parts of the World [24-29]. Recent studies have estimated the current prevalence of AMD from around 1.47%[30] in the United States, where they predict that 2.95 million people will have AMD by 2020, to as high as 3.5% in United Kingdom[9]. There more than half of the registered blindness (54.5%) is due to degeneration at the macula or posterior pole. A cumulative 5-year person specific incidence of 1% was found which climbed to 2.8% after 10-year follow-up. Klein et al found a more conservative 2.1% incidence [31].

AMD is subdivided into two types. The most common form is dry AMD (85%). Secondly there is wet or Choroidal revascularization (CNV). Because of the variety of pathogenic factors underlying AMD, there is no straightforward treatment for AMD and posing the question whether we are dealing with completely deferent diseases[32]. Studies to date however find the same genetics behind both forms of AMD[33].

Current treatment methods focus mainly on the CNV form of AMD. Injections with Bevacizumab and Ranibizumab monthly for 2 years resulted in up to 2 lines of visual acuity gain on a standardized eye chart compared to 2 or more lines loss in control groups [34-37]. But these, like other therapies as thermal laser surgery[32] and photodynamic therapy [38], only modulate the disease and have their limitations. Autologous retinal pigment epithelium (RPE) transplantation is one feasible surgery technique but studies still show varying degrees of in results and complications [39, 40]. While this technique also showed positive effect on geographical atrophy AMD, a dry from of AMD, also with the same restrictions as for CNV [41], there still is no established treatment for the dry form of AMD.

Blue light hazard (excitation peak 440nm) was reported to have major impact on photoreceptor and RPE function inducing photochemical damage and cellular apoptosis, leading to retinal degeneration in animal study [10]. The current believe is that lutein and zeaxanthin accumulated in the macular region can help in the prevention of AMD by absorbing this blue light and protecting the retina from oxidative stress by neutralizing free radicals [11, 42-46]. With the lipid matrix of the egg yolk being a proven vehicle for the efficient absorption of dietary lutein [12, 47-49] and zeaxanthin [50], it is be possible to increase plasma levels and macula levels[51]. This may lead to therapeutic levels and be able to control or prevent AMD [11, 43, 45, 52].

Omega-3 fatty acids have in some studies been shown to prevent AMD [53, 54], when combined with a low omega-6 fatty acid diet [55], but data to date remains inconclusive [56]. The possible protective nature of omega-3 is believed to work by regulating inflammatory and immune response in the retina, repairing damaged cells, and improving endothelial cell function [53].

In this study we hope to show that the consumption of enriched eggs or egg products will lead to an increase in the macular pigment optical density. This we hope can then slow the progression of AMD (both dry and wet) and maybe prevent its onset.

2. OBJECTIVES

Primary Objective: Assessing macular pigments' spatial distribution and the changes after consumption of modified eggs compared to normal eggs.
Secondary Objective(s): Assessing changes in retinal morphology, visual acuity and contrast sensitivity. We will also evaluate lipoprotein metabolism in relation to daily egg consumption.

3. STUDY DESIGN

This will be a randomized placebo-controlled trial. The total study time will be two years of which 3 months actual trial and follow up time. Every individual with have 3 measuring point at set interval. At every measuring point (days 1, 45 and 90) these subjects will undergo 6 different non-invasive measuring techniques. These are the mean visual acuity test using the Early Treatment Diabetic Retinopathy Study (ETDRS)-chart, contrast sensitivity using the Pelli-Robson chart, Scanning Laser Ophthalmoscope (SLO), Optical Coherence Tomography (OCT) and Heterochromatic Flicker Photometry (HFP) and the Reflectometer. A questionnaire will be taken at the beginning of the trail. The invasive part of the study involves blood sampling at all three times, measuring the serum concentration of lutein, zeaxanthin, omega-3 and lipoprotein using the HPLC analysis.
At the beginning and twice more during the trial period, test subject will keep a nutrition diary for one day.

4. STUDY POPULATION

4.1 Population (base)

One hundred (100) healthy subjects 18 years and older will be included.

4.2 Inclusion criteria

- No history of ARM or AMD
- 18 years and older
- Non-smoker
- No ocular media opacity
- Uses no nutritional supplements containing Lutein, Zeaxanthin or Omega-3
- BMI < 30
- No known cardiovascular disease

4.3 Exclusion criteria

- Diabetes
- Other known eye disease
- Known lipid metabolism disease
- Blood lipid level modifiers (e.g. Statin)
- Known allergy to eggs or egg products

4.4 Sample size calculation

Inname van 10 mg luteïne uit een voedingssupplement laat een toename zien in de hoeveelheid macula pigment van 5% per maand [57]. Inname van 20 mg luteïne laat een vergelijkbare toename zien [58]. De hoeveelheid luteïne in één ei per dag ligt maximaal op 1 mg. Echter de beschikbaarheid van luteïne in eieren is veel groter dan in voedingssupplementen. We verwachten daarom op zijn minst een toename van 3% per 4 weken, hetgeen resulteert in 9% toename in 3 maanden.
De hoeveelheid macula pigment zal worden bepaald met de Macula Pigment Reflectometer, die een meetfout heeft van 7% [21]. We willen drie groepen met elkaar vergelijken. Om een significant verschil in macula pigment dichtheid van 9% aan te kunnen tonen zijn dan 18 proefpersonen per groep bij α=0.5 (Bonferroni correctie 0.017) en β=0.1 nodig [59]. Uitgaande van 10% uitval, komen we op 20 proefpersonen per groep.

Opmerking [TB1]: *Intake of 10 mg lutein from supplements induces a 5% monthly increase in MPOD [57]. Intake of 20 mg induces a similar increase [58]. Although the amount of lutein per egg is much less, the bioavailability is higher . We therefore anticipated a 3% monthly increase in MPOD, resulting in a 9% MPOD increase over the study period. The repeatability of the QuantifEYE is 11.7%. With α=0.05 and β=0.1 this yielded 18 subjects per group [59]. Taking into account a 10% dropout we included 20 subjects per group.*

TREATMENT OF SUBJECTS

4.5 Investigational product/treatment

Each individual will consume one egg or egg product daily for a period of 90 days. These will be either modified or control eggs. The expiration time for eggs is 28 days. By using boiled eggs stored in a refrigerator this shelf live can be extended to 8 weeks.
The buttermilk product, which is pasteurized, tested for cooled shelf live for a period of 6 weeks, and 4 weeks at room temperature.

Testing shows the following concentrations for the eggs:

In egg (mg/egg)	Lutein Mg per egg yolk		Zeaxanthine Mg per egg yolk	
	mean	St dev	mean	St dev
Control	0,16778	0,008655	0,08504	0,001692
Lutein	0,921422	0,105767	0,137318	0,013953
Zeaxanthine	0,1743	0,014454	0,487303	0,031019

The omega-3 concentration is the same for all eggs and is 200 ± 10mg.

4.6 Use of co-intervention (if applicable)

Not applicable

4.7 Escape medication (if applicable)

Not applicable.

5. INVESTIGATIONAL MEDICINAL PRODUCT

Lutein, zeaxanthin and omega3 fatty acid are not medication but supplements. They are pro-vitamin and as such readily available at any drugstore. Furthermore, they are all naturally occurring supplements which are present in many vegetables (lutein and zeaxanthin) and fish (omega-3 fatty acid).

Testing shows the following concentrations for the eggs:

In egg (mg/egg)	Lutein Mg per egg yolk		Zeaxanthine Mg per egg yolk	
	mean	St dev	mean	St dev
Control	0,16778	0,008655	0,08504	0,001692
Lutein	0,921422	0,105767	0,137318	0,013953
Zeaxanthine	0,1743	0,014454	0,487303	0,031019

The omega-3 concentration is the same for all eggs and is 200 ± 10mg.

5.1 Name and description of investigational medicinal product

Not applicable

5.2 Summary of findings from non-clinical studies

Not applicable

5.3 Summary of findings from clinical studies

After 6 months of supplementation 10 mg (containing 5% of zeaxanthin) the serum level of lutein increased from 210 to 1000 nM/L and for zeaxanthin from 56 to 95 nM/l[60]. No toxicity was observed at 2.5, 5.0 or 10.0mg daily in persons over the age of 60 [16].
The potential antithrombotic effect of Omega-3 fatty acids may theoretically increase risk for bleeding, this theory has however no clinical support [15].

5.4 Summary of known and potential risks and benefits

To date there are no known risk or potential risk from the consumption of lutein [16, 17], zeaxanthin[16] or omega-3 fatty acid [15]. There have been gastrointestinal complaints been associated with omega-3 fatty acids intake but with no serious consequences[61]. Increased Cholesterol levels, induced by daily egg consumption has to date not been shown to be a significant factor for heart disease risk[62]. There is however evidence that lutein and zeaxanthin may protect against age-related macula degeneration [11, 42, 43, 45, 46, 58]. There is also sufficient data supporting the protective role of omega-3 on cardiovascular diseases [63] and it’s independent association with lower levels of anti-inflammatory markers [64]. There is also indication of its beneficial effects on a myriad of other disorders [65].

5.5 Description and justification of route of administration and dosage

These supplements will be consumed in the form of an egg or egg product daily. Research have shown egg yolk as a effective and efficient vehicle for the absorption of lutein and zeaxanthin [12, 50].

5.6 Dosages, dosage modifications and method of administration

Test subjects will receive one of four different products to consume during the trial period of 90 days. These will all contain 0.921422 mg/egg (st dev ± 0.105767) of Lutein, 0.487303 mg/egg (st dev. ± 0.031019) of Zeaxanthin, and 200 (± 10) mg of Omega-3 fatty acids. The dosage is stated as approximately as there are either eggs or egg-products which are produced naturally and as so the concentration will vary from between eggs. The concentration in the egg product will be more uniformly ass these are made from a single batch, which will give less variation in concentration than for those subjects consuming the egg itself. These products has to be taken daily for 90 days at lunch time as either an egg or a buttermilk drink containing one egg yolk.

5.7 Preparation and labeling of Investigational Medicinal Product

The products supplied to the University of Maastricht for the human intervention study are from normal commercial production. They are produced in a HACCP certified poultry farms and an EU certificated egg processing facility. All appropriate food safety standards (Productschap Diervoederwetgeving en GMP-regeling) are rigorously applied to the ingredients, the processing and the packaging. As a HACCP poultry farm and an EU certificated egg processing facility, production records are retained and available for inspection as required under the EU legislation. We have produced some test batches for determining the shelf life time of the product. From the above, it goes without saying that these products are entirely suitable and safe for human consumption.
The modified eggs will be produced by Nelissen Pluimvee using feeds prepared by Research Diet Services B.V. in Wijk bij Duurstede. Coppelmans in Waalre will prepare the egg product.
The feeds for producing the enriched eggs will be formulated and produced within the legal requirements for animal feed. The use of lutein and zeaxanthin is regulated under EU regulation 1831/2003. The dosage of lutein and zeaxanthin in feed will not exceed the legal limit of 80 ppm in animal feed. For omega3 fatty acids is a component of some ingredients like linseed meal and fish oil. These products are not limited for use as feed ingredient. Nowadays these feeds are already in use to produce omega3 fatty acids enriched eggs.
Within the legal requirements no extra labelling of the investigational product is necessary. The normal expiry time for eggs is 28 days. By using boiled eggs stored in a refrigerator this shelf live can be extended to 8 weeks.
The buttermilk product is tested for shelf live for a period of 6 weeks.

5.8 Drug accountability

The eggs will be produced by Nelissen Pluimvee. The boiled eggs will be prepared by Nelissen Pluimvee and distributed by the office of Newtricious. The eggs will be packed in board or plastic containers. The products will be transported to the Human Biology distribution facility where test subjects can collect their products.

The buttermilk drink will be prepared by Coppelmans and packed in 100 ml bottles. The products will be transported to the Human Biology distribution facility where test subjects can collect their products.

Returned products from test subjects will be returned to Newtricious, which will assure the destruction of the remaining products.

6. METHODS

6.1 Study parameters/endpoints

6.1.1 Main study parameter/endpoint

Macular pigment optical density: the amount of measurable macular pigment in the macula region.

6.1.2 Secondary study parameters/endpoints (if applicable)

Blood lutein, zeaxanthin, omega-3, and lipid levels. Change in visual acuity and contrast sensitivity.

6.1.3 Other study parameters (if applicable)

Iris colour, diet, daily sun exposure time, alcohol consumption, anti cholesterol medication, vitamin supplement, and presence of intra ocular lens.

6.2 Randomisation, blinding and treatment allocation

Randomisatie zal middels loting plaatsvinden. Deze zullen een volgnummer krijgen welke zal bepalen in welke groep ze terecht komen. Er vind geen blindering plaats.

Opmerking [TB2]: *Randomization will take place by drawing lots. This will generate a tracking number, which will determine in which group a particular subject will fall. There will be no blinding.*

6.3 Study procedures

Blood sampling

1. een lipiden profiel bestaande uit fasting totaal cholesterol, HDL cholesterol en Triaccylglycerol. Hieruit kunnen we dan LDL cholesterol berekenen mbv de Friedewald formule.
2. apoA-I en apoB
3. een klinisch safety profile (ALAT, ASAT, creatinine, gamma GT, alkalisch phosphatase, bilirubine)
4. luteine en zeaxanthine
5. markers die een low grade inflammatory profile aangeven zoals hs-CRP
6. markers die endotheel functie weergeven zoals sICAM, sVCAM sE-selectin, en MCP-1
7. markers voor oxidatieve schade (zou moeten dalen door al die aox die je geeft) bv MDA en oxLDL
8. omega-3

We hebben hiervoor nodig per meetmoment:
1 serum buis 10 ml
1 EDTA buis 10 ml

The researcher will take the blood using two vacuum viles using the appropriate needles. These will be stored after being processed in containers in an ultra low temperature freezer (minus 80º Celsius) situated at the Human Biology department.

Heterochromatic Flicker Photometry

The non researched eye is blindfolded. Subjects take place on a chair and look through the ocular, with the eye being researched, at a white background where 3 black dots are depicted. They are initially instructed to look ad the centremost dot and to press the button on the remote as soon as it starts to flicker. They are told that the colour of the dots will change after the fifth time and that the test is not finished. A distinctive tone will alert them at the start of the second measurement. They are now instructed to look at and fixate on either the left or the right dot (depending on the measured eye). Subject are now meant to press again on the button of the remote as soon as they see the centremost dot flickering again, this in order to measure the peripheral field of vision. After these last five repetitions the same tone will indicate the end of the test.

SLO

Test subject take place on a chair in front of the SLO and rest head on a chinrest with both temples again a cushioned stay. The test eye is aligned with the laser beam and the raster is focused on the retina. In the centre of the raster there appears a cross. Subjects are clearly asked to keep focusing on the cross which centres the raster onto the macula. All this can be followed on a monitor linked to the SLO. The researcher now takes a series of pictures of the macula using different colours. After about 3 minutes the test is completed. Subjects may come from behind the SLO. If they like they can see the pictures taken.

Reflectometer

The patient will be seated with both forehead and chin supported. They will be instructed to look straight forward onto a bright white spot. The researcher will record the difference between the light entering and leaving the eye. The subject will not notice any of this. The difference will give us an adequate estimate of the amount of Macular Pigment.

Optical Coherence Tomography

The patient will be seated with both forehead and chin supported on a rest. The OCT is aligned in front of the eye being tested. Subjects will be asked whether they see a green light, which they have to focus on. If that is not the case, an external fixation point, in the mode of a blinking LED, will be used. They will be warned that the apparatus will get quite close to the eye without actually touching it. We will point out that it is normal to see a moving light source during the test, this is the recording device. Clear instructions will be given as to when they may blink, this in order to obtain clear images. After about 4 minutes when the test is finished they can see the results if they like to.

Visual acuity test with the ETDRS-chart

Subject will be seated at 5metre distance from the ETDRS-chart. This under optimal contrast sensitivity conditions (room dimly lid, and chart adequately lid by an other light source). Depending on the line and amount of mistakes subject made while reading the

chart with one eye closed at a time, their visual acuity will be drafted in a logarithmic-value.

Contrast sensitivity

Subjects will be seated with both chin and head supported by the chin and head rest looking straight ahead to a computer monitor at 2.25 meter away. One eye, the one not being tested, is blindfolded. The researcher will then instruct the subject to look at the monitor onto which a circle is displayed with clearly visible vertical stripes in black and white (and all the greyscale tones in between). These will alternate in a sinusal rhythm. The researcher can then change the contrast manually. Subjects are then instructed to say “yes” and “no” as to whether or not they see the stripes. In this manner the researcher will get ever closer to the threshold value for contrast sensitivity of the test subject. Once this value is reached the computer automatically changed to the next spatial frequency (subjects will be test on a total of 6 frequencies). Each time the researcher will adapt the contrast until the threshold is reached. This will continue for up to 10 minutes, depending on visual acuity and contrast sensitivity op subjects.

Questionnaire

This is a short list of question regarding personal information, iris colour, refractory correction, diet, and lifestyle. This will be in Dutch. Subjects will also be asked to keep a nutrition diary. A nutrition diary will be filled for three days during the trial.

Informed consent

Every subject participating in this trial will sign a standard form consenting to the trial and stating their understanding of the implication of their participation. This will be in Dutch.

6.4 Withdrawal of individual subjects

Subjects can leave the study at any time for any reason if they wish to do so without any consequences. The investigator can decide to withdraw a subject from the study for urgent medical reasons. Data collected from subject will be destroyed immediately.

6.4.1 Specific criteria for withdrawal (if applicable):

Not applicable

6.5 Replacement of individual subjects after withdrawal

Not applicable

6.6 Follow-up of subjects withdrawn from treatment

As we are measuring change over time and as there is no adverse effect to be expected subject who withdraw will not be followed on. They will be encouraged thou to contact us in case of any doubt.

6.7 Premature termination of the study

If for any reason we are to believe that the eggs or egg products are having serious adverse effects on the test subject, the study will be immediately terminated, however unlikely such an event may be. Our measurements involves proven techniques which

have already shown in many studies not to produce any SAE's, however, if such an event is to happen the study will also be terminated.

In either case test subjects will be informed in writing explaining the reasons for and possible action they need to take because of the serious adverse effects. All available measurement data will de destroyed, this in keeping with the subjects' anonymity.

7. SAFETY REPORTING

7.1 Section 10 WMO event

In accordance to section 10, subsection 1, of the WMO, the investigator will inform the subjects and the reviewing accredited METC if anything occurs, on the basis of which it appears that the disadvantages of participation may be significantly greater than was foreseen in the research proposal. The study will be suspended pending further review by the accredited METC, except insofar as suspension would jeopardise the subjects' health. The investigator will take care that all subjects are kept informed.

7.2 Adverse and serious adverse events

If any adverse of serious adverse event is to happen, subjects will be immediately informed, both by phone (this being faster) as in writing. They will then be given instruction to immediately stop using the trail products and if need be consult their physician, unless they require immediate medical attention in which case they will be instructed to report to the nearest emergency unit which will be update by us on the condition and necessary treatment.

Adverse events are defined as any undesirable experience occurring to a subject during a clinical trial, whether or not considered related to the investigational drug. All adverse events reported spontaneously by the subject or observed by the investigator or his staff will be recorded.

A serious adverse event is any untoward medical occurrence or effect that at any dose results in death;

- is life threatening (at the time of the event);
- requires hospitalisation or prolongation of existing inpatients' hospitalisation;
- results in persistent or significant disability or incapacity;
- is a congenital anomaly or birth defect;
- is a new event of the trial likely to affect the safety of the subjects, such as an unexpected outcome of an adverse reaction, lack of efficacy of an IMP used for the treatment of a life threatening disease, major safety finding from a newly completed animal study, etc.

All SAEs will be reported to the accredited METC that approved the protocol, according to the requirements of that METC.

7.3 Follow-up of adverse events

All adverse events will be followed until they have abated, or until a stable situation has been reached. Depending on the event, follow up may require additional tests or medical procedures as indicated, and/or referral to the general physician or a medical specialist.

7.4 Data Safety Monitoring Board (DSMB)

Not applicable

8. STATISTICAL ANALYSIS

The statistical software package SPSS v15.0 en Stata/SE v8.2 will be used for data analysis. The students' T-test and ANOVA will be used to evaluate possible differences in continues variables between men and women, between subject with high and low fruit and vegetables intake en between subject with different iris colour.

The Chi-square test will be used to asses difference between baseline characteristics between groups (sex, iris colour, fruit and vegetable intake). If variables are not distributed normally they will be converted into Log-values.

In order to estimate changes in macular pigment optical density and retinal thickness over time, the General Linear Model (GML) will be applied for repeated measurements, with time as within subject factor and age, and iris colour as covariant.

9. ETHICAL CONSIDERATIONS

9.1 Regulation statement

This study will be conducted according to the principles of the Declaration of Helsinki last amended in 2000 including the latest Note of Clarification of Tokyo, 2004 and in accordance with the Medical Research Involving Human Subjects Act (WMO).

9.2 Recruitment and consent

Folders will be displayed at different location at the University of Maastricht and in the waiting room of the polyclinic office of the ophthalmology department at the University Hospital of Maastricht (AZM) asking those interested to contact us. There will also be advertised in the local newspapers.

Subjects suitable for participation will be informed orally informed by the researcher of the purpose and implications of this trial. They will receive a brief explanation of the test performed and what they are to expect both from the trail as from the researcher. They will be given a 3 day windows to think over their decision. They are free to ask anything they want to know. Once they agree to participate and they conform to the inclusion criteria, they will sign an informed consent form.

9.3 Objection by minors or incapacitated subjects (if applicable)

Not applicable

9.4 Benefits and risks assessment, group relatedness

With this trial we hope to provide a friendlier and effective manner of consuming lutein, zeaxanthin and omega-3 fatty acid in order to improve the quality of life of thousands, if not millions of people.

This trial will be performed on healthy test subject. In theory this may help prevent the development of AMD in these subjects. However, because of the shortness of the trial period this is no realistic expectation.

The benefits of this study flows from the knowledge gained as to whether or not macular pigment optical density increase or improvement in visual acuity is seen after consumption of modified eggs or egg products. If that is the case, this may lead to simple adjustments to daily routine by means of daily egg of egg product consumption which may help delay the progress of AMD or even help in preventing it.

As until know there are no known surgical or medical therapy for AMD and the current injection techniques only modify the disease and are no cure (at a cost of about €1000,00 per treatment), this seem a more friendly way of treating or maybe even preventing AMD. As there are no known risks involved in consuming these supplements, we believe the benefits outnumber the risks. An independent physician has been appointed for an objective view.

9.5 Compensation for injury

The University Hospital of Maastricht (azM) has issued an insurance for subjects participating in this trial falling within the scope of the WMO in accordance with the legal requirements of article 7 of the WMO and Medical (Human Subjects) Compulsory Insurance Decree of 23 June 2003. This has been attained at Lloyd's London, Marketform Holdings Ltd, London EC 3M5EA.

The sponsor/investigator has a liability insurance which is in accordance with article 7, subsection 6 of the WMO.

The sponsor (also) has an insurance which is in accordance with the legal requirements in the Netherlands (Article 7 WMO and the Measure regarding Compulsory Insurance for Clinical Research in Humans of 23rd June 2003). This insurance provides cover for damage to research subjects through injury or death caused by the study.

1. € 450.000,-- (i.e. four hundred and fifty thousand Euro) for death or injury for each subject who participates in the Research;
2. € 3.500.000,-- (i.e. three million five hundred thousand Euro) for death or injury for all subjects who participate in the Research;
3. € 5.000.000,-- (i.e. five million Euro) for the total damage incurred by the organisation for all damage disclosed by scientific research for the Sponsor as 'verrichter' in the meaning of said Act in each year of insurance coverage.

The insurance applies to the damage that becomes apparent during the study or within 4 years after the end of the study.

9.6 Incentives

Test subject will receive full compensation for transportation fee for every test moment. An additional monetary incentive will be given for every completed part of the study, totalling €100.00.They will not be compensated in any manner other than that.

10. ADMINISTRATIVE ASPECTS AND PUBLICATION

10.1 Handling and storage of data and documents

Every measurement will be coded and as such handled by the researcher. Every subject's data will be code as follow: land code/ subject number/ data number, i.e. NL-02-1 (The Netherlands, test subject number 2, and data from 1st measurement). Only the researcher and research coordinator will have access to these information codes key. The data will be kept at least till the end of the analysis and subsequent publication. Blood samples will be stored for at least 1 year till they have all been analyzed. All data will be kept in a secured computer and freezer (in the case of blood samples) so as to prevent any unauthorized person from gaining access to information which might reveal test subjects identity.

10.2 Amendments

Amendments are changes made to the research after a favourable opinion by the accredited METC has been given. All amendments will be notified to the METC that gave a favourable opinion.

10.3 Annual progress report

The sponsor/investigator will submit a summary of the progress of the trial to the accredited METC once a year. Information will be provided on the date of inclusion of the first subject, numbers of subjects included and numbers of subjects that have completed the trial, serious adverse events/ serious adverse reactions, other problems, and amendments.

10.4 End of study report

The investigator will notify the accredited METC of the end of the study within a period of 8 weeks. The end of the study is defined as the last patient's last visit.

In case the study is ended prematurely, the investigator will notify the accredited METC, including the reasons for the premature termination.

Within 18 months after the end of the study, the investigator/sponsor will submit a final study report with the results of the study, including any publications/abstracts of the study, to the accredited METC. Data collected will be kept for 5 years after termination of the

study after which they will be destroyed. This in case samples have to be re-analyzed.

10.5 Public disclosure and publication policy

In contract opgesteld met Newtricious B.V., staat vermeld onder artikel 6 en artikel 7, het volgende:
Artikel 6. Kennisinbreng en rechten op kennis en Onderzoekresultaten

6.1 Kennis die voor aanvang van het Onderzoek reeds in eigendom was van een Partij alsmede kennis die is gegenereerd door een Partij buiten de strekking van het Onderzoek, blijft ondervermindert eigendom van die Partij.

6.2 Partijen zullen in het kader van het uit deze samenwerking voortvloeiende Onderzoek kennis uitwisselen.

6.3 Partijen hebben het recht gebruik te maken van de Onderzoeksresultaten die door alle Partijen in het kader van het Onderzoek worden gegenereerd, met het doel om het Onderzoek uit te voeren.

Opgebouwde kennis en onderzoeksresultaten voortvloeiende uit dit onderzoek komen in eigendom van Newtricious.

6.5 Newtricious bepaalt of en wanneer er een octrooi wordt aangevraagd met als inhoud de resultaten uit het in dit project uitgevoerde onderzoek.

Artikel 7. Publicatie van Onderzoekresultaten

Onderzoeker en Instituut hebben het recht tot publicatie van de resultaten van de metingen. Kopieën van abstracts, papers, mondelinge presentaties, manuscripten of publicaties of enige informatie mbt data of resultaten verkregen of gegenereerd gedurende de Studie en als resultaat van deze overeenkomst, zullen minstens 30 dagen voor indiening ter publicatie, voorgelegd worden aan Newtricious. Newtricious zal de voorgelegde publicatie slechts beoordelen met het oog op de bescherming van bedrijfsgeheimen en/of het veilig stellen van mogelijke octrooiaanvragen, en eventuele suggesties doen tot aanpassingen, welke in redelijkheid door Onderzoeker/Instituut zullen worden overgenomen.
Het auteursrecht op publicaties door de Onderzoeker behoort exclusief toe aan de Onderzoeker en zal niet worden overgedragen aan Newtricious.

Opmerking [TB3]: In the contract with Newtricious BV is listed under Articles 6 and 7, the following:
Article 6. Knowledge Contribution and rights to knowledge and research results

6.1 Knowledge that was already a available to a Party before the Research and knowledge generated by a Party outside the scope of research, remains property of that Party.

6.2 The parties will exchange knowledge in the context of the research resulting from this collaboration.

6.3 The parties have the right to use results generated by this research, that were generated by all Parties to in order to perform the reserach

Accumulated knowledge and research results arising from this study will be owned by Newtricious.

6.5 Newtricious determines if and when there will be patent applications from out the results of the research carried out in this project.

Opmerking [TB4]: Article 7. Publication of Research Results

Researcher Institute and have the right to publish the results of the measurements. Copies of abstracts, papers, oral presentations, manuscripts or publications or any information about dates and results obtained or generated during the study and as a result of this agreement, will be at least 30 days prior to submission for publication, submitted to Newtricious. Newtricious will assess the submitted publication only with a view to the protection of trade secrets and / or securing of any patent, and any suggestions for changes, which in fairness will be taken by Researcher / Institute
The copyright on publications by the investigator belongs exclusively to the Researcher and will not be transferred to Newtricious.
Further publication policy will be in accordance with to the CCMO-statement (see attachment)

11. REFERENCES

1. Foran, S., J.J. Wang, and P. Mitchell, *Causes of visual impairment in two older population cross-sections: the Blue Mountains Eye Study.* Ophthalmic Epidemiol, 2003. **10**(4): p. 215-25.
2. Bunce, C. and R. Wormald, *Leading causes of certification for blindness and partial sight in England & Wales.* BMC Public Health, 2006. **6**: p. 58.
3. Rosenberg, T. and F. Klie, *The incidence of registered blindness caused by age-related macular degeneration.* Acta Ophthalmol Scand, 1996. **74**(4): p. 399-402.
4. Leibowitz, H.M., et al., *The Framingham Eye Study monograph: An ophthalmological and epidemiological study of cataract, glaucoma, diabetic retinopathy, macular degeneration, and visual acuity in a general population of 2631 adults, 1973-1975.* Surv Ophthalmol, 1980. **24**(Suppl): p. 335-610.
5. Bamashmus, M.A., B. Matlhaga, and G.N. Dutton, *Causes of blindness and visual impairment in the West of Scotland.* Eye, 2004. **18**(3): p. 257-61.
6. The Eye Diseases Prevalence Research, G., *Causes and Prevalence of Visual Impairment Among Adults in the United States.* Arch Ophthalmol, 2004. **122**(4): p. 477-485.
7. Owen, C.G., et al., *How big is the burden of visual loss caused by age related macular degeneration in the United Kingdom?* Br J Ophthalmol, 2003. **87**(3): p. 312-317.
8. Olmedilla, B., et al., *Lutein, but not [alpha]-tocopherol, supplementation improves visual function in patients with age-related cataracts: a 2-y double-blind, placebo-controlled pilot study.* Nutrition, 2003. **19**(1): p. 21-24.
9. Wang, J.J., et al., *Ten-Year Incidence and Progression of Age-Related Maculopathy: The Blue Mountains Eye Study.* Ophthalmology, 2007. **114**(1): p. 92-98.
10. Algvere, P.V., J. Marshall, and S. Seregard, *Age-related maculopathy and the impact of blue light hazard.* Acta Ophthalmol Scand, 2006. **84**(1): p. 4-15.
11. Landrum, J.T. and R.A. Bone, *Lutein, zeaxanthin, and the macular pigment.* Arch Biochem Biophys, 2001. **385**(1): p. 28-40.
12. Chung, H.-Y., H.M. Rasmussen, and E.J. Johnson, *Lutein Bioavailability Is Higher from Lutein-Enriched Eggs than from Supplements and Spinach in Men.* J. Nutr., 2004. **134**(8): p. 1887-1893.
13. Novello, D., et al., *[Egg: concepts, analyses and controversies in the human health].* Arch Latinoam Nutr, 2006. **56**(4): p. 315-20.
14. McNamara, D.J., *The Impact of Egg Limitations on Coronary Heart Disease Risk: Do the Numbers Add Up?* J Am Coll Nutr, 2000. **19**(90005): p. 540S-548.

15. Bays, H.E., *Safety Considerations with Omega-3 Fatty Acid Therapy.* The American Journal of Cardiology, 2007. **99**(6, Supplement 1): p. S35-S43.
16. Rosenthal, J.M., et al., *Dose-ranging study of lutein supplementation in persons aged 60 years or older.* Invest Ophthalmol Vis Sci, 2006. **47**(12): p. 5227-33.
17. Shao, A. and J.N. Hathcock, *Risk assessment for the carotenoids lutein and lycopene.* Regulatory Toxicology and Pharmacology, 2006. **45**(3): p. 289-298.
18. Jean, B., A. Frohn, and H.J. Thiel, *[Laser scanning in ophthalmology].* Fortschr Ophthalmol, 1990. **87**(2): p. 158-67.
19. Klingbeil, U., *Safety aspects of laser scanning ophthalmoscopes.* Health Phys, 1986. **51**(1): p. 81-93.
20. Delori, F.C., et al., *Macular pigment density measured by autofluorescence spectrometry: comparison with reflectometry and heterochromatic flicker photometry.* J Opt Soc Am A Opt Image Sci Vis, 2001. **18**(6): p. 1212-30.
21. van de Kraats, J., et al., *Fast assessment of the central macular pigment density with natural pupil using the macular pigment reflectometer.* J Biomed Opt, 2006. **11**(6): p. 064031.
22. Wolfs, R.C., et al., *Risk of acute angle-closure glaucoma after diagnostic mydriasis in nonselected subjects: the Rotterdam Study.* Invest Ophthalmol Vis Sci, 1997. **38**(12): p. 2683-7.
23. Patel, K.H., et al., *Incidence of acute angle-closure glaucoma after pharmacologic mydriasis.* Am J Ophthalmol, 1995. **120**(6): p. 709-17.
24. Iwase, A., et al., *Prevalence and Causes of Low Vision and Blindness in a Japanese Adult Population: The Tajimi Study.* Ophthalmology, 2006. **113**(8): p. 1354-1362.e1.
25. Xu, L., et al., *Causes of Blindness and Visual Impairment in Urban and Rural Areas in Beijing: The Beijing Eye Study.* Ophthalmology, 2006. **113**(7): p. 1134.e1-1134.e11.
26. Buch, H., T. Vinding, and N.V. Nielsen, *Prevalence and causes of visual impairment according to World Health Organization and United States criteria in an aged, urban Scandinavian population: The Copenhagen City Eye Study.* Ophthalmology, 2001. **108**(12): p. 2347-2357.
27. Hsu, W.-M., et al., *Prevalence and causes of visual impairment in an elderly Chinese population in Taiwan : The Shihpai Eye Study.* Ophthalmology, 2004. **111**(1): p. 62-69.
28. Buch, H., et al., *Prevalence and causes of visual impairment and blindness among 9980 Scandinavian adults: The Copenhagen City Eye Study.* Ophthalmology, 2004. **111**(1): p. 53-61.
29. Buch, H., et al., *The prevalence and causes of bilateral and unilateral blindness in an elderly urban Danish population. The Copenhagen City Eye Study.* Acta Ophthalmol Scand, 2001. **79**(5): p. 441-9.

30. The Eye Diseases Prevalence Research, G., *Prevalence of Age-Related Macular Degeneration in the United States.* Arch Ophthalmol, 2004. **122**(4): p. 564-572.

31. Klein, R., et al., *Ten-year incidence and progression of age-related maculopathy: The Beaver Dam eye study.* Ophthalmology, 2002. **109**(10): p. 1767-1779.

32. Bylsma, G.W. and R.H. Guymer, *Treatment of age-related macular degeneration.* Clin Exp Optom, 2005. **88**(5): p. 322-34.

33. Scholl, H.P., et al., *An update on the genetics of age-related macular degeneration.* Mol Vis, 2007. **13**: p. 196-205.

34. Brown, G.C., et al., *A Value-Based Medicine Comparison of Interventions for Subfoveal Neovascular Macular Degeneration.* Ophthalmology, 2007. **In Press, Corrected Proof**.

35. Rosenfeld, P.J., et al., *Ranibizumab for Neovascular Age-Related Macular Degeneration.* N Engl J Med, 2006. **355**(14): p. 1419-1431.

36. Liew, G. and P. Mitchell, *Ranibizumab for neovascular age-related macular degeneration.* N Engl J Med, 2007. **356**(7): p. 747-8; author reply 749-50.

37. Gillies, M.C. and T.Y. Wong, *Ranibizumab for neovascular age-related macular degeneration.* N Engl J Med, 2007. **356**(7): p. 748-9; author reply 749-50.

38. Soubrane, G. and N.M. Bressler, *Treatment of subfoveal choroidal neovascularisation in age related macular degeneration: focus on clinical application of verteporfin photodynamic therapy.* Br J Ophthalmol, 2001. **85**(4): p. 483-495.

39. MacLaren, R.E., et al., *Autologous Transplantation of the Retinal Pigment Epithelium and Choroid in the Treatment of Neovascular Age-Related Macular Degeneration.* Ophthalmology, 2007. **114**(3): p. 561-570.e2.

40. Treumer, F., et al., *Autologous retinal pigment epithelium-choroid sheet transplantation in age related macular degeneration: morphological and functional results.* Br J Ophthalmol, 2007. **91**(3): p. 349-353.

41. Joussen, A.M., et al., *Autologous Translocation of the Choroid and Retinal Pigment Epithelium in Patients with Geographic Atrophy.* Ophthalmology, 2007. **114**(3): p. 551-560.

42. O'Connell, E., et al., *Macular carotenoids and age-related maculopathy.* Ann Acad Med Singapore, 2006. **35**(11): p. 821-30.

43. Krinsky, N.I., J.T. Landrum, and R.A. Bone, *BIOLOGIC MECHANISMS OF THE PROTECTIVE ROLE OF LUTEIN AND ZEAXANTHIN IN THE EYE.* Annual Review of Nutrition, 2003. **23**(1): p. 171-201.

44. Landrum, J.T., R.A. Bone, and M.D. Kilburn, *The macular pigment: a possible role in protection from age-related macular degeneration.* Adv Pharmacol, 1997. **38**: p. 537-56.

45. Rapp, L.M., S.S. Maple, and J.H. Choi, *Lutein and Zeaxanthin Concentrations in Rod Outer Segment Membranes from Perifoveal and Peripheral Human Retina.* Invest. Ophthalmol. Vis. Sci., 2000. **41**(5): p. 1200-1209.
46. Beatty, S., et al., *Macular pigment and age related macular degeneration.* Br J Ophthalmol, 1999. **83**(7): p. 867-877.
47. Leeson, S. and L. Caston, *Enrichment of eggs with lutein.* Poult Sci, 2004. **83**(10): p. 1709-12.
48. Calvo, M.M. and iacute, *Lutein: A Valuable Ingredient of Fruit and Vegetables.* Critical Reviews in Food Science and Nutrition, 2005. **45**(7): p. 671 - 696.
49. Granado, F., B. Olmedilla, and I. Blanco, *Nutritional and clinical relevance of lutein in human health.* Br J Nutr, 2003. **90**(3): p. 487-502.
50. Handelman, G.J., et al., *Lutein and zeaxanthin concentrations in plasma after dietary supplementation with egg yolk.* Am J Clin Nutr, 1999. **70**(2): p. 247-51.
51. Bone, R.A., et al., *Distribution of Lutein and Zeaxanthin Stereoisomers in the Human Retina.* Experimental Eye Research, 1997. **64**(2): p. 211-218.
52. Beatty, S., et al., *Macular Pigment and Risk for Age-Related Macular Degeneration in Subjects from a Northern European Population.* Invest. Ophthalmol. Vis. Sci., 2001. **42**(2): p. 439-446.
53. Seddon, J.M., S. George, and B. Rosner, *Cigarette Smoking, Fish Consumption, Omega-3 Fatty Acid Intake, and Associations With Age-Related Macular Degeneration: The US Twin Study of Age-Related Macular Degeneration*. 2006. p. 995-1001.
54. Hodge, W.G., et al., *Evidence for the effect of omega-3 fatty acids on progression of age-related macular degeneration: a systematic review.* Retina, 2007. **27**(2): p. 216-21.
55. Seddon, J.M., et al., *Dietary Fat and Risk for Advanced Age-Related Macular Degeneration*. 2001. p. 1191-1199.
56. Hodge, W.G., et al., *Efficacy of [omega]-3 Fatty Acids in Preventing Age-Related Macular Degeneration: A Systematic Review.* Ophthalmology, 2006. **113**(7): p. 1165-1173.
57. Berendschot, T.T.J.M., et al., *Influence of Lutein Supplementation on Macular Pigment, Assessed with Two Objective Techniques.* Invest. Ophthalmol. Vis. Sci., 2000. **41**(11): p. 3322-3326.
58. Landrum, J.T., et al., *A one year study of the macular pigment: the effect of 140 days of a lutein supplement.* Exp Eye Res, 1997. **65**(1): p. 57-62.
59. Dupont, W.D. and W.D. Plummer, *PS power and sample size program available for free on the internet.* Controlled Clinical Trials, 1997. **18**(3): p. 274.

60. Khachik, F., et al., *The effect of lutein and zeaxanthin supplementation on metabolites of these carotenoids in the serum of persons aged 60 or older.* Invest Ophthalmol Vis Sci, 2006. **47**(12): p. 5234-42.
61. Nair, S.S.D., et al., *Prevention of Cardiac Arrhythmia by Dietary (n-3) Polyunsaturated Fatty Acids and Their Mechanism of Action*. 1997. p. 383-393.
62. McNamara, D.J., *Eggs and heart disease risk: perpetuating the misperception.* Am J Clin Nutr, 2002. **75**(2): p. 333-334.
63. Harper, C.R. and T.A. Jacobson, *Beyond the Mediterranean diet: the role of omega-3 Fatty acids in the prevention of coronary heart disease.* Prev Cardiol, 2003. **6**(3): p. 136-46.
64. Ferrucci, L., et al., *Relationship of Plasma Polyunsaturated Fatty Acids to Circulating Inflammatory Markers*. 2006. p. 439-446.
65. Lee, S., et al., *Current clinical applications of omega-6 and omega-3 fatty acids.* Nutr Clin Pract, 2006. **21**(4): p. 323-41.
